# Nd: YAG laser in modern dentistry: Advances in periodontal, endodontic, and soft tissue therapeutics

**DOI:** 10.6026/973206300220379

**Published:** 2026-01-31

**Authors:** Salina Manandhar, Ladan Rafiei, Pantea Kaviandost, Min Thu Linn, Nidhiswi Yeruva, Harpreet Kaur

**Affiliations:** 1 Department of Dental Surgery, Rangpur Community Medical and Dental College and Hospital, Rangpur, Bangladesh, South Asia; 2 Department of Dental Surgery, Zahedan Medical University of Dental Science, Zahedan, Iran; 3 Department of Dental Surgery, Tbilisi State Medical University, Tbilisi, Georgia; 4 Department of Dental Surgery, University of Dental Medicine, Yangon, Myanmar; 5 Department of Dental Surgery, Kamineni Institute of Dental Sciences, Nalgonda, India; 6 Department of Dental Surgery, Baba Farid University of Health and Sciences, Punjab, India

**Keywords:** Nd:YAG laser, periodontal therapy, endodontics, soft tissue surgery, dentin hypersensitivity

## Abstract

The Nd: YAG laser has emerged as a versatile tool in dentistry due to its selective interaction with pigmented tissues and its relatively deep penetration into oral structures. Therefore, it is of interest to review the core mechanisms, key clinical applications in periodontics, endodontics, soft-tissue surgery and dentin hypersensitivity, as well as the safety principles governing responsible use. Evidence indicates significant reductions in bacterial load, improved hemostasis and patient comfort when parameters are calibrated appropriately. Important limitations include thermal risk, cost and a learning curve, reinforcing the need to integrate lasers with conventional techniques. Overall, Nd: YAG technology can enhance minimally invasive dental care when applied within evidence-based protocols.

## Background:

The conceptual foundation for understanding how dental tissues respond to external stimuli dates back to the hydrodynamic theory of dentin sensitivity, which describes how movement of fluid within dentinal tubules triggers a pain response [1]. This early insight into the interaction between external forces and tooth structure paved the way for exploring methods that could modify or control these responses, eventually leading to the consideration of lasers as therapeutic tools. Alongside the growing interest in precision dental treatments, research into the biological effects of temperature on oral structures demonstrated that exceeding certain thermal thresholds could cause irreversible tissue damage. In an *in vivo* study, it was evident that sustained temperatures above 47°C can result in bone injury [2]. This finding became important for evaluating heat-generating devices such as lasers, underscoring the need for strict operational control to ensure safety. The introduction of lasers into dentistry marked a major advance in minimally invasive techniques. Early reviews highlighted the ability of dental lasers to cut, coagulate and disinfect tissues with reduced trauma compared to conventional instruments [3]. Among the available laser systems, the neodymium-doped yttrium aluminum garnet (Nd: YAG) laser, operating at a wavelength of 1064 nm, quickly drew attention for its unique absorption profile. This wavelength interacts strongly with pigmented chromophores such as hemoglobin and melanin, enabling selective targeting of vascular and inflamed tissues. One of the earliest clinical uses of Nd: YAG lasers were in treating dentin hypersensitivity. Researchers demonstrated that laser irradiation seals dentinal tubules by thermally modifying the surface, thus reducing fluid flow and alleviating pain [4]. This principle-selective alteration of tissue microstructure through controlled thermal energy-later formed the basis for broader dental applications. In microbiological studies, the Nd: YAG laser has proven to be particularly effective against pigmented bacteria, which are common in periodontal and endodontic infections. Its photothermal energy disrupted bacterial cell walls and altered protein structures, producing significant reductions in microbial load [5]. The capacity of the 1064 nm wavelength to penetrate deeper into soft tissues than many other dental lasers also meant it could reach microorganisms hidden beyond the immediate surface. Further investigation into its clinical use confirmed that the Nd: YAG laser could access areas within the root canal system that were inaccessible to traditional irrigation and instrumentation [6]. This ability to combine deep tissue penetration with selective microbial targeting positioned the Nd: YAG laser as a versatile instrument for both soft tissue management and endodontic disinfection. The combination of precise energy delivery, selective tissue interaction and proven antimicrobial efficacy led to the establishment of the Nd: YAG laser as an important addition to the dentist's toolkit. With these fundamental mechanisms understood, researchers began to evaluate their role in more complex procedures-most notably in periodontal therapy, where tissue preservation and infection control are paramount. This exploration forms the focus of the next section [5].

## Periodontal applications of ND: YAG lasers:

Building on the understanding of its basic mechanisms, the Nd: YAG laser found a prominent role in periodontal therapy due to its ability to selectively target diseased tissue while preserving healthy structures. Early reviews emphasized that laser treatment in periodontics could result in reduced bleeding, enhanced bacterial elimination and patient comfort improvements compared to conventional techniques [7]. Clinical applications in pediatric dentistry, particularly for soft tissue removal, provided further insight into its precision and hemostatic capabilities. Nd: YAG laser excision procedures demonstrated minimal bleeding, reduced postoperative discomfort and precise control over tissue removal [8]. These qualities translated effectively into adult periodontal care, where controlled soft tissue debridement is critical for long-term stability. The laser's potential in managing dentin hypersensitivity-originally applied in non-periodontal cases-proved equally valuable in patients with gingival recession and exposed root surfaces. Sealing dentinal tubules through thermal modification offered both immediate pain relief and protection against further bacterial ingress [9]. This preventive benefit complemented its primary role in active periodontal therapy. However, temperature regulation remained a critical consideration. Excessive laser output could raise root surface and surrounding bone temperatures beyond safe thresholds, risking irreversible damage [10]. This finding reinforced the importance of calibrated parameters and appropriate operator training to ensure safety while achieving therapeutic goals. Long-term management of periodontal patients also relies on controlling hypersensitivity, which can interfere with oral hygiene practices and overall treatment compliance. Early work on dentin hypersensitivity provided a foundational understanding for applying Nd: YAG therapy in maintenance phases [11]. By reducing discomfort, patients could more effectively perform plaque control measures, contributing to sustained periodontal stability. Clinical trials demonstrated that adjunctive use of Nd: YAG lasers with scaling and root planing (SRP) significantly improved treatment outcomes. In a controlled study, patients receiving combined therapy achieved greater probing depth reduction, increased clinical attachment gain and less bleeding on probing compared to SRP alone [12]. Building on periodontal success, Moritz *et al.* demonstrated that Nd:YAG laser irradiation could significantly reduce intracanal bacteria, supporting its use in root canal therapy where effective disinfection of complex anatomical spaces is a persistent challenge [7]. The following section explores how the Nd:YAG laser has been integrated into endodontic treatment strategies.

## Endodontic applications of ND: YAG lasers:

Following its success in periodontal therapy, the Nd: YAG laser became an attractive tool for endodontics due to its unique combination of deep penetration and selective antimicrobial effects. Endodontic infections often involve complex root canal anatomies with lateral canals, isthmuses and dentinal tubules that can harbor microorganisms inaccessible to mechanical instruments or conventional irrigants. Early investigations demonstrated that Nd: YAG laser irradiation could significantly reduce bacterial loads in infected root canals, particularly targeting pigmented bacteria such as Porphyromonas gingivalis and Prevotella intermedia [13]. Further research compared the bactericidal effects of Nd: YAG and other laser systems. It was found that the 1064 nm wavelength could penetrate dentinal tubules more effectively than visible light lasers, reaching depths of up to 1000 µm and delivering lethal energy to bacteria without causing structural damage to the surrounding dentin [14]. This depth of penetration was especially advantageous against pathogens residing beyond the immediate canal surface. In addition to its antimicrobial properties, the Nd: YAG laser proved effective for smear layer removal. The smear layer-composed of dentin debris, pulp remnants and microorganisms-can block the penetration of sealers and medicaments into dentinal tubules. Studies demonstrated that Nd: YAG laser irradiation could remove or modify this layer, improving the adhesion and sealing ability of obturation materials [15]. Enhanced sealing reduces the risk of reinfection and improves the long-term prognosis of root canal treatments. Another significant advantage is the laser's ability to alter dentin structure. By melting and resolidifying the dentin surface, Nd: YAG irradiation can occlude tubules, reduce permeability and potentially strengthen the treated surface. This modification is not only beneficial for endodontic sealing but also contributes to reduced postoperative sensitivity [16]. Clinical protocols combining Nd: YAG laser therapies with conventional irrigants, such as sodium hypochlorite, have shown synergistic effects. The laser's thermal energy disrupts bacterial biofilms, while the irrigant flushes out debris and dissolved tissue remnants. This dual approach enhances canal cleanliness and maximizes microbial reduction [17]. Importantly, these protocols must adhere to specific energy settings to prevent heat damage to periapical tissues, aligning with safety considerations established in earlier thermal studies. Beyond disinfection, the Nd: YAG laser has applications in vital pulp therapy. Its ability to achieve hemostasis quickly and stimulate odontoblast-like cell activity makes it suitable for pulpotomy and direct pulp capping. The photo-bio modulatory effects of sub-ablative energy doses can promote reparative dentin formation, aiding in the preservation of pulp vitality [18]. Overall, the Nd: YAG laser's role in endodontics is well-supported by both laboratory and clinical research. Its ability to combine deep antimicrobial action, smear layer management and dentin modification offers significant advantages over conventional methods. These benefits have encouraged exploration of its use in broader soft tissue procedures within dentistry-an area that demonstrates the laser's versatility beyond periodontal and endodontic cares [5]. The following section examines its applications in oral soft tissue management.

## ND: YAG laser applications in soft tissue procedures:

The adaptability of the Nd: YAG laser extends beyond hard tissue and endodontic applications, finding a significant role in oral soft tissue surgery. Its capacity for precise cutting, coagulation and hemostasis makes it a valuable tool in both routine and complex procedures. Early studies emphasized that when used within safe energy parameters, the Nd: YAG laser could provide efficient disinfection without causing surface carbonization or structural compromise, even in deeper tissues [19]. This level of control is especially important when working in highly vascularized oral regions. One of the most recognized soft tissue protocols incorporating Nd: YAG technology is the Laser-Assisted New Attachment Procedure (LANAP), designed for periodontal regeneration without traditional flap surgery. Clinical reports documented reduced bleeding, preservation of connective tissue and improved healing, reinforcing the idea that laser-assisted methods could replace or supplement conventional surgical techniques in select cases [20]. In addition to regenerative periodontal procedures, Nd: YAG lasers have been studied for their bactericidal effectiveness against resilient organisms such as Enterococcus faecalis-a pathogen also implicated in persistent periapical infections. *in vitro* experiments demonstrated that laser irradiation could achieve significant bacterial kill rates even in the presence of complex root anatomy, providing a foundation for its combined hard and soft tissue applications [21]. Its value in soft tissue management is further highlighted in the treatment of conditions such as hypertrophic frenum, gingival hyperplasia and operculum removal in orthodontic cases. Procedures utilizing Nd: YAG lasers often result in minimal intraoperative bleeding and reduced postoperative discomfort, with many cases requiring no sutures. This is consistent with findings from diode and Nd: YAG laser studies that emphasized their efficient coagulation abilities [22]. From an educational standpoint, early reviews of laser use in dentistry underscored the importance of understanding both the physical properties of each wavelength and the clinical protocols for safe application. The Nd: YAG laser's low absorption in water and high affinity for pigmented tissues position it uniquely among dental lasers, allowing deeper tissue penetration than CO_2_ or Er: YAG systems [23]. Perhaps most importantly, its ability to achieve predictable hemostasis is not just a matter of surgical convenience but also one of patient safety and comfort. By controlling bleeding and reducing the need for mechanical suturing, postoperative recovery can be expedited. These benefits have been observed in clinical practice and in experimental models, where Nd: YAG lasers demonstrated reduced postoperative swelling and infection risk [24]. Collectively, these features make the Nd: YAG laser a versatile instrument in soft tissue dentistry, where precision and patient comfort are paramount. Its proven safety under controlled parameters, combined with predictable clinical outcomes, has solidified its role alongside more traditional instruments. The benefits of faster healing, less postoperative pain and consistent results make Nd: YAG laser a valuable option that significantly impacts patient quality of life and presents a unique opportunity for minimally invasive laser treatment [34]. Moving from the realm of surgical interventions, another promising area of Nd: YAG application lies in the management of dentin hypersensitivity.

## ND: YAG laser management of dentin hypersensitivity:

Dentin hypersensitivity is a common dental complaint characterized by a brief, sharp pain in response to stimuli such as temperature changes, tactile pressure, or chemical exposure. While it does not pose a direct threat to tooth vitality, it can significantly affect oral function and patient comfort [1]. Nd: YAG laser therapy has emerged as a promising minimally invasive approach, capitalizing on its ability to modify dentin structure and seal exposed tubules. Studies investigating Nd: YAG's bactericidal effects have provided indirect benefits for hypersensitivity management. By eliminating pigmented and non-pigmented bacteria implicated in dentin surface biofilm, laser treatment reduces inflammation and helps create a cleaner substrate for tubule sealing [25]. This antimicrobial capability complements the laser's thermal action, which induces melting and re-solidification of peritubular and inter-tubular dentin, leading to tubule occlusion [26]. An *in vitro* study evaluating the irradiation effects of Nd: YAG and diode lasers on infected root canals showed that the Nd: YAG system effectively disrupted bacterial membranes and altered dentin permeability [27]. These same principles underpin its role in hypersensitivity reduction, where decreasing fluid movement within dentinal tubules is central to Brännström's hydrodynamic theory. The clinical literature also supports Nd: YAG therapy for reducing hypersensitivity following periodontal or surgical interventions. A review of its application in dentistry highlighted the wavelength's deep penetration and affinity for pigmented structures, enabling targeted thermal effects without excessive water absorption or risk of superficial overheating [28]. This precise interaction with tooth substrates ensures both safety and comfort during treatment. Beyond its role in hypersensitivity, Nd: YAG therapy has been assessed in non-surgical periodontal protocols. A controlled clinical trial evaluated its use in combination with scaling and root planning, reporting significant improvements in clinical attachment level and reduced bleeding on probing compared to conventional therapy alone [29]. While this study focused primarily on periodontal health, the reduced sensitivity reported by participants underscores the crossover benefits of laser use. Further expanding the evidence base, research has shown that Nd: YAG laser treatment can improve outcomes in moderate to severe periodontitis cases, particularly when combined with other laser wavelengths or adjunctive antimicrobial measures [30]. Patients often experience not only enhanced periodontal healing but also relief from hypersensitivity symptoms associated with root exposure. The broad applicability of Nd: YAG lasers-from microbial control to dentin surface modification-positions them as a valuable alternative or adjunct to topical desensitizing agents. While topical products may require frequent reapplication and provide variable results, laser therapy often delivers longer-lasting effects after a single session. This advantage, combined with minimal discomfort during application, makes it an attractive option for both patients and clinicians [31]. With strong support from laboratory, clinical and review studies, Nd: YAG lasers have proven to be effective for hypersensitivity management while delivering additional periodontal and antimicrobial benefits [32]. The final section will address safety considerations, potential limitations and the clinical protocols necessary to maximize the benefits of this versatile technology while minimizing associated risks.

## Safety considerations, limitations and conclusion:

While Nd: YAG lasers have demonstrated substantial versatility in periodontal, endodontic, soft tissue and hypersensitivity applications, their clinical benefits are closely tied to correct usage and adherence to safety protocols. The wavelength's deep penetration and strong affinity for pigmented tissues offer advantages but also increase the risk of unintended thermal effects if parameters are not carefully controlled. Excessive energy delivery can lead to collateral damage, including pulp injury, periodontal ligament trauma, or even alveolar bone necrosis [33]. The wavelength's high penetration depth means that tissues beyond the intended target may absorb energy, necessitating precise angulation, controlled exposure time and the use of pulsed rather than continuous-wave settings. Clinical guidelines recommend the use of protective eyewear for all individuals in the operatory, as Nd: YAG lasers emit near-infrared light that poses significant ocular hazards [34]. Another limitation lies in the system's selectivity. While Nd: YAG lasers excel against pigmented bacteria, their efficacy against non-pigmented species such as Enterococcus faecalis can be comparatively reduced [35]. This underscores the importance of integrating laser therapy with conventional mechanical and chemical disinfection methods, rather than relying on it as a sole treatment modality. In surgical contexts, the Nd: YAG laser's ability to coagulate and vaporize tissue without excessive bleeding is beneficial, but it may be less efficient for hard tissue removal compared to erbium-based systems. This makes it less suitable for procedures like cavity preparation or complete calculus removal in heavily mineralized deposits [36]. In such cases, the Nd: YAG is best used as a complementary instrument rather than a standalone alternative. Economic factors also affect its widespread adoption. Nd: YAG units are typically more expensive than diode or CO_2_ systems, with higher maintenance costs and a steeper learning curve. The need for operator certification and ongoing training can pose additional barriers, particularly for smaller dental practices. Nonetheless, the long-term benefits in patient satisfaction and clinical outcomes can justify these investments in well-selected cases [35].

## Conclusion:

The Nd: YAG laser is a versatile tool that improves treatment precision and, when incorporated into a carefully designed treatment plan, can raise the standard of care in periodontal therapy, endodontic disinfection, soft tissue surgery and the management of hypersensitivity. Its effectiveness, however, relies on the clinician's expertise, adherence to evidence-based protocols and maintaining realistic expectations of its strengths and limitations. As research continues to refine optimal settings, explore synergistic combinations with other modalities and expand its clinical indications, the Nd: YAG laser is likely to play an increasingly important role in modern dentistry.
